# *Notes from the Field: Candida auris* and Carbapenemase-Producing Organism Prevalence in a Pediatric Hospital Providing Long-Term Transitional Care — Chicago, Illinois, 2019

**DOI:** 10.15585/mmwr.mm6934a5

**Published:** 2020-08-28

**Authors:** Tristan D. McPherson, Kelly A. Walblay, Elissa Roop, David Soglin, Ann Valley, Latania K. Logan, Snigdha Vallabhaneni, Stephanie R. Black, Massimo Pacilli

**Affiliations:** ^1^Epidemic Intelligence Service, CDC; ^2^Communicable Disease Program, Chicago Department of Public Health, Illinois; ^3^La Rabida Children’s Hospital, Chicago, Illinois; ^4^Wisconsin State Laboratory of Hygiene; ^5^Rush University Medical Center, Chicago, Illinois; ^6^Division of Healthcare Quality Promotion, National Center for Emerging and Zoonotic Infectious Diseases, CDC.

*Candida auris* is an emerging fungal pathogen that is frequently drug-resistant; *C. auris* can be difficult to identify, and it has been associated with outbreaks in health care settings.* The first case of *C. auris* in Chicago, Illinois, was identified in May 2016 (*1*). Additional cases continue to be reported, particularly in high-acuity, postacute–care facilities (*1*), and spread of *C. auris* within this type of facility has been documented nationwide (*2*). To monitor local trends in the prevalence of *C. auris*, point prevalence surveys (PPSs) have been conducted in Chicago since August 2016 (*1*). In addition to *C. auris*, a high prevalence of carbapenemase-producing organisms (CPOs) has also been described in Chicago long-term acute-care hospitals since 2010 (*3*). *C. auris* and CPOs can colonize persons over prolonged periods and, because of antimicrobial resistance, cause invasive infections with limited treatment options (*2*,*3*). Co-colonization with these organisms has been identified (*4*). Adults in long-term acute-care hospitals are at increased risk for acquiring *C. auris* and CPOs because of serious underlying medical conditions, extended lengths of stay, presence of indwelling medical devices, and frequent health care worker contact (*3*,*4*). As of June 2019, among residents of Chicago’s four long-term acute-care hospitals, the median prevalences of colonization with *C. auris* and CPO were 31% and 24%, respectively (Chicago Department of Public Health, personal communication, January 3, 2020). Although prevalence among adults is well characterized, prevalence of *C. auris* colonization has not been described among pediatric populations in Chicago, and limited data exist on CPO colonization among children outside of intensive care units (*5*).

To assess *C. auris* and CPO colonization among children, in August 2019, the Chicago Department of Public Health conducted a PPS in a 49-bed pediatric hospital providing long-term transitional care for patients leaving pediatric intensive care units. All hospitalized patients were included unless parental consent could not be obtained. Presence and type of medical devices (i.e., gastrostomy tubes, tracheostomies, mechanical ventilators, and central venous catheters) and lengths of stay were documented for all hospitalized patients. Specimens collected for testing consisted of composite bilateral axillary and inguinal swabs for *C. auris* and rectal swabs for CPO testing. The Wisconsin State Laboratory of Hygiene tested all specimens. Real-time polymerase chain reaction (PCR) assays were used to detect *C. auris* DNA and the carbapenemase genes *bla*_KPC_, *bla*_NDM_, *bla*_VIM_, *bla*_OXA-48_, and *bla*_IMP_ (Xpert Carba-R Assay, Cepheid). All axillary and inguinal swabs were processed by real-time PCR and culture to identify *C. auris*. For CPOs, culture was attempted on real-time PCR-positive specimens. Among all 29 hospitalized patients, 25 (86%) were screened for *C. auris* and CPOs. Two rectal specimens were unsatisfactory and produced invalid CPO test results. Patient census was matched to the Illinois extensively drug resistant organism (XDRO) registry to identify previous reports of *C. auris* and CPO colonization or infection. Facility prevalence of *C. auris* and CPOs was calculated as the number of patients with a PPS-related specimen that was positive or a previous report of these organisms in the XDRO registry divided by the facility census count provided by the facility on the day of PPS.

Among the 29 hospitalized patients, median age was 1.2 years (range = 26 days–17.4 years; interquartile range [IQR] = 289 days–2.6 years), 26 (90%) had a gastrostomy tube, 24 (83%) had a tracheostomy, 20 (69%) required mechanical ventilation, and three (10%) had a central venous catheter. Median length of stay was 35 days (IQR = 13–71 days). No patient had a previous report of *C. auris* or CPO. No patient specimens were positive for *C. auris*, and a specimen from one patient was positive for *bla*_OXA-48_, yielding a facility prevalence for CPOs of 3.4%. No organism was recovered from the specimen that tested positive for *bla*_OXA-48_.

This PPS is the first documented screening for *C. auris* colonization in a transitional care pediatric hospital in the United States. Despite a high prevalence of *C. auris* and CPOs among patients in adult health care settings of similar acuity in the region, *C. auris* was not identified and CPOs were rare at this pediatric hospital. Biannual assessment of this facility is planned. Because this PPS includes only one facility in a region, additional evaluations in similar pediatric health care settings need to be conducted to improve understanding of *C. auris* and CPO prevalence in this population.

## Footnotes

* https://www.cdc.gov/fungal/candida-auris/index.html.
